# 厦门市居民肺癌死亡与减寿趋势分析及预测

**DOI:** 10.3779/j.issn.1009-3419.2016.02.04

**Published:** 2016-02-20

**Authors:** 艺兰 林, 啸青 伍, 田泉 林

**Affiliations:** 1 361021 厦门，厦门市疾病预防控制中心 Xiamen Center for Disease Control and Prevention, Xiamen 361021, China; 2 361021 厦门，福建医科大学预防医学专业教学基地 Teaching Base of Preventive Medicine, Fujian Medical University, Xiamen 361021, China; 3 361021 厦门，厦门大学公卫学院预防医学教学基地 School of Preventive Medicine Teaching Base, Xiamen University, Xiamen 361021, China; 4 361003 厦门，厦门市妇幼保健院 Xiamen Municipal Maternal and Child Health Hospital, Xiamen 361003, China

**Keywords:** 肺肿瘤, 死亡率, 潜在寿命损失, 预测, GM（1, 1）模型, Lung neoplasms, Mortality, Potential years of life lost, Prediction, GM(1, 1) model

## Abstract

**背景与目的:**

近年来肺癌发病率和死亡率不断上升，已成为我国恶性肿瘤的首位死因。本研究旨在探讨厦门市居民肺癌死亡和减寿的变化趋势，以期为厦门市肺癌综合防治工作提供依据。

**方法:**

收集整理2005年-2014年厦门市居民肺癌死亡资料计算死亡率、平均减寿年数（average potential life lost, AYLL）、死亡率年均变化百分比等评价指标，用GM（1, 1）模型对死亡率和AYLL进行预测。

**结果:**

2005年-2014年，厦门市居民肺癌死亡率28.58/10万，年均上升4.86%，男性死亡率是女性的2.90倍；AYLL为7.8年，存在下降趋势。GM（1, 1）模型预测值与实际值平均相对误差2.16%-8.83%，预测2015年-2019年肺癌死亡率和AYLL值均有所上升。

**结论:**

厦门市肺癌死亡率逐年升高，未来肺癌死亡率和人均减寿数都有上升趋势，应重视肺癌的预防控制工作。

## Abstract (en)

**Background and objective:**

In recent years, the incidence and mortality of lung cancer is rising. It has become the leading cause of death of malignant tumors in China. The aim of this study is to explore the trend of mortality and years of life lost due to lung cancer in residents in Xiamen, so as to provide the basis data on preventing lung cancer in Xiamen.

**Methods:**

The data of residents in Xiamen dying of lung cancer from 2005 to 2014 was collected and cleared up to calculate the evaluation indexes including the mortality rate, the average potential life lost (AYLL), and the average percentage change (APC) of mortality rate. GM(1, 1) model was used to predict the future mortality and AYLL.

**Results:**

From 2005 to 2014, the average mortality rate of lung cancer in residents in Xiamen was 28.58 per 100, 000 persons, of which in male was 2.90 times as that in female. The APC was 4.86%. The AYLL, which was 7.8 years, had decline trend from 2005 to 2014. The mean absolute percentage errors between observed values and fitted values were 2.16%-8.83%. The mortality rate and AYLL of lung cancer in residents in Xiamen would increase from 2015 to 2019.

**Conclusion:**

The mortality of lung cancer increased year by year in Xiamen. There are both increasing trend of mortality and years of life lost in future. So we should pay more attentions on preventing and curing of lung cancer.

肺癌是常见的恶性肿瘤之一，近年来伴随人口老龄化的加剧、人们生活环境和生活方式的改变，其发病率和死亡率不断上升，2011年全国新发病例约65万，死亡病例约52万，已成为我国恶性肿瘤的首位死因^[1]^。肺癌也是厦门市居民最常见的恶性肿瘤，发病率和死亡率均居厦门市恶性肿瘤发病谱和死因谱的首位^[2, 3]^。为进一步了解肺癌对厦门市居民健康的影响，本研究对厦门市近十年来的肺癌死亡和减寿情况及趋势进行分析和预测，以期为厦门市肺癌综合防治工作提供依据。

## 1 资料与方法

### 1.1 资料来源

2005年1月1日-2014年12月31日厦门市户籍人口肺癌死亡资料来自厦门市死因监测系统。厦门市2005年死因监测已覆盖全市六个区，并统一采用ICD-10进行疾病分类。以2010年全国人口普查数据为标准人口。各年人口学数据由厦门市公安局提供。

### 1.2 统计指标和方法

用Deathreg 2002软件录入死亡资料，之后导出至Excel 2007进行整理分析、制表，用SAS 9.2软件进行统计分析。分析指标包括：肺癌死亡率、标化死亡率（standardized mortality rate, SMR）、潜在寿命损失（potential years of life lost, PYLL）、平均减寿年数（average potential life lost, AYLL）和寿命损失率（rate of potential years of life lost, PYLLR）。SMR=(∑Nsipi)/*Ni*，PYLL=∑[L-(xi+0.5)]×di，AYLL=PYLL/*n*，PYLLR=(PYLL/*N*)×1, 000，其中Nsi为第i个年龄组的标准人口数，pi为第i个年龄组的实际死亡率，*Ns*为标准人口总人口数，L为期望寿命，定为75岁，xi和di为i年龄组组中值和组距，*n*为肺癌实际死亡总数，*N*为实际人群总人口数。死亡率的变化趋势用死亡率年均变化百分比（average annual percent change, APC）衡量，APC采用线性回归法计算，对APC的检验转为对斜率a的*t*检验，公式如下y=ax+b，APC(%)=(e^a^-1)×100；其中x为年份，y为各年死亡率的自然对数值。*P* < 0.05为差异有统计学意义。

### 1.3 死亡率及减寿情况预测方法

死亡率和AYLL值采用GM（1, 1）模型进行预测。用累加法生成数据，计算m=x_t+1_/x_t_进行事前检验，其中x_t_为t年发病率；阈值范围为[e^-2/(n+1)^, e^2/(n+1)^]，其中*n*为用于预测的数据个数，若计算结果不完全在阈值范围内，则尝试对数据进行平方根转换后建模；事后检验采用后验差比值C进行评价，C=S_e_/S_x_，其中S_x_为数据标准差，S_e_为残差标准差；C≥0.65模型为不合格，0.5≤C < 0.65模型为勉强合格、0.35≤C < 0.5模型为合格、C < 0.35模型为优。不合格模型不能用于预测，若模型不合格，则尝试减少用于预测的数据个数进行建模。用平均相对误差（mean absolute percentage error, MAPE）对预测精度进行评价，MAPE(%)=∑︱(x_t_-x_t_′)/x_t_′︳*100/*n*，其中x_t_为实际值，x_t_′为预测值。

## 2 结果

### 2.1 肺癌死亡总体情况

2005年-2014年厦门市居民因肺癌死亡5, 112例，死亡率为28.58/10万，标化死亡率30.66/10万；其中男性死亡3, 806例，死亡率为42.45/10万，标化死亡率48.47/10万；女性死亡1, 306例，死亡率为14.64/10万，标化死亡率14.65/10万。男性死亡率是女性的2.90倍，男性标化死亡率是女性的3.31倍。各年男性死亡率和标化死亡率始终高于女性。无论男性、女性，肺癌死亡率均随着年龄的增长而升高，在75岁-79岁年龄组达到高峰，高峰值分别为417.29/10万、121.39/10万。2005年-2014年厦门市居民肺癌死亡率均存在平稳上升趋势，年均上升4.86%（*t*=9.00, *P* < 0.05），其中男性、女性分别上升5.55%和3.34%（*t*分别为12.87、2.95，*P*均 < 0.05）（表 1）。

**1:** 2005年-2014年厦门市肺癌死亡率和标化死亡率（1/10万） Mortality rate and standard mortality rate of lung cancer in Xiamen from 2005 to 2014 (1/10^5^)

Year	Male			Female			Total	
	Mortality	SMR		Mortality	SMR		Mortality	SMR
2005	31.63	37.20		11.88	12.14		21.86	24.09
2006	33.56	39.64		13.03	13.33		23.41	25.92
2007	38.74	45.68		14.75	15.36		26.87	29.63
2008	38.19	42.70		15.42	15.70		26.89	28.73
2009	38.69	45.86		12.13	12.38		25.48	28.03
2010	45.03	52.47		14.32	14.40		29.72	32.38
2011	43.83	50.04		14.11	14.49		28.94	31.21
2012	47.18	54.25		14.32	14.27		30.69	33.11
2013	49.25	53.61		15.98	15.31		32.52	33.45
2014	53.22	57.17		19.02	18.12		35.98	36.67
Total	42.45	48.47		14.64	14.65		28.58	30.66
SMR: standardized mortality rate.								

### 2.2 肺癌所致减寿情况

2005年-2014年厦门市居民因肺癌所致PYLL为39, 884人年，AYLL为7.80年，PYLLR为2.23‰；其中男性因肺癌所致PYLL为29, 868人年，AYLL为7.85年，PYLLR为3.33‰；女性因肺癌所致PYLL为10, 016人年，AYLL为7.67年，PYLLR为1.12‰。各年男性因肺癌所致PYLL和PYLLR均明显高于女性，而AYLL男性与女性差别不大。无论男女，肺癌所致AYLL均存在下降趋势，而PYLLR存在上升趋势，2014与2005年相比，因肺癌所致AYLL下降13.78%，PYLLR上升41.75%（表 2）。

**2:** 2005年-2014年厦门市居民因肺癌所致寿命损失情况 Life lost due to lung cancer in Xiamen from 2005 to 2014

Year	Male				Female				Total		
	PYLL (year)	PYLLR (‰)	AYLL (year/person)		PYLL (year)	PYLLR (‰)	AYLL (year/person)		PYLL (year)	PYLLR (‰)	AYLL (year/person)
2005	2, 189.5	2.83	8.94		777.0	1.03	8.63		2, 966.5	1.94	8.86
2006	2, 206.0	2.78	8.29		796.5	1.03	7.89		3, 002.5	1.91	8.18
2007	2, 900.0	3.40	8.79		875.5	1.05	7.12		3, 775.5	2.24	8.33
2008	3, 074.5	3.48	9.12		1, 277.0	1.47	9.53		4, 351.5	2.48	9.24
2009	2, 437.5	2.73	7.07		717.5	0.81	6.71		3, 155.0	1.78	6.98
2010	2, 622.0	2.94	6.52		1, 052.5	1.19	8.29		3, 674.5	2.06	6.95
2011	3, 066.5	3.32	7.57		1, 094.5	1.18	8.35		4, 161.0	2.25	7.76
2012	3, 502.5	3.66	7.77		735.0	0.76	5.33		4, 237.5	2.21	7.19
2013	3, 680.0	3.71	7.54		1, 291.0	1.29	8.07		4, 971.0	2.50	7.67
2014	4, 189.5	4.15	7.80		1, 399.5	1.37	7.18		5, 589.0	2.75	7.64
Total	29, 868.0	3.33	7.85		10, 016.0	1.12	7.67		39, 884.0	2.23	7.80
PYLL: potential years of life lost; PYLLR: rate of potential years of life lost; AYLL: average potential life lost.											

### 2.3 死亡率和AYLL值预测结果

模型所用的数据和建模结果见表 3。六个模型经事后检验，C值在0.20-0.63之间，模型均可用于预测。预测值与实际值平均相对误差除女性AYLL较大，为8.83%外，余MAPE在2.16%-3.84%间。预测2015年-2019年厦门市居民肺癌死亡率在36.13/10万-43.40/10万之间，肺癌所致AYLL在7.87年-8.49年间；与2014年相比，2019年肺癌死亡率增加20.62%，肺癌所致AYLL增加0.85年；男女肺癌死亡率和AYLL值均有所上升，APC斜率检验均有统计学意义（*t*在192-4, 160之间，*P*均 < 0.000, 1）；与2014年相比，2019年女性肺癌死亡率增加39.70%，男性肺癌死亡率增加27.83%（表 4）。

**3:** GM(1, 1)模型建模参数和预测精度 Modeling parameters and prediction accuracy of GM(1, 1) model

Dependent variable	Data used in building model	α^*^	μ^*^	C	MAPE (%)
Mortality rate					
Male	2005-2014	-0.051, 81	32.163, 3	0.20	2.52
Female	2008-2014	-0.077, 74	10.544, 2	0.38	3.84
Total	2005-2014	-0.045, 85	22.369, 7	0.29	3.45
AYLL					
Male	2008-2014^*^^*^	-0.013, 46	2.566, 1	0.40	3.11
Female	2011-2014^*^^*^	-0.067, 99	2.155, 5	0.63	8.83
Total	2008-2014^*^^*^	-0.009, 51	2.607, 6	0.31	2.16
^*^: model parameter; ^**^: Data was conversed by square root.					

**4:** GM(1, 1)模型预测结果 Prediction result of GM(1, 1) model

Year	Male			Female			Total	
	Mortality rate (1/10^5^)	AYLL (year)		Mortality rate (1/10^5^)	AYLL (year)		Mortality rate (1/10^5^)	AYLL (year)
2015	55.30	8.10		19.47	8.91		36.13	7.87
2016	58.24	8.32		21.04	10.20		37.83	8.02
2017	61.34	8.54		22.74	11.69		39.60	8.17
2018	64.60	8.78		24.58	13.39		41.46	8.33
2019	68.03	9.02		26.57	15.34		43.40	8.49

## 3 讨论

肺癌已成为我国恶性肿瘤的首位死因，2011年全国肺癌死亡病例约52万，死亡率达39.27/10万，占全人群总死亡的1/4^[4]^。本研究结果显示，2005年-2014年厦门市居民肺癌年均死亡率为28.58/10万，低于全国平均水平，与福建省2007年-2011年的肺癌年均死亡率29.35/10万接近^[5]^，说明厦门市肺癌死亡率还处在全国中下水平，但全市肺癌死亡率上升趋势明显，年均上升4.86%，高于全国平均年增长率2.79%^[4]^，2012年开始超过肝癌，成为厦门市居民恶性肿瘤的首位死因，表明肺癌对厦门市居民健康的危害正日益加剧，迫切需要采取合理有效措施遏制或减缓肺癌死亡上升趋势。本研究还显示各年男性死亡率和标化死亡率始终高于女性，且死亡率均随着年龄的增长而升高，在75岁-79岁年龄组达到高峰，与屈若等^[6]^的研究结果一致，分析原因可能与男性吸烟率高、职业暴露机会多和厦门市人口结构逐步老龄化等因素有关，提示应把男性老年人群作为重点干预和保护对象。

在分析疾病负担时，PYLL、AYLL和PYLLR纳入居民死亡的年龄因素，将死亡人数和死亡年龄相结合，更能客观地反映疾病对人群死亡的影响。其中AYLL侧重强调某种疾病导致该病患者寿命损失年数，值越大说明该疾病影响的重点人群年龄越轻^[7]^；而PYLLR侧重考虑某疾病对全人群寿命的影响，除受死者年龄影响外，还与全人群该病死亡水平有关。本研究结果显示，2005年-2014年厦门市居民因肺癌所致PYLL为39, 884人年，AYLL为7.80年，比肺癌所致福建省AYLL少2.11年；PYLLR为2.23‰，也低于福建省2010年肺癌所致的PYLLR 3.36‰^[7]^，说明与全省水平相比，肺癌对厦门市居民早死的影响还较低。同时，2005年-2014年厦门居民因肺癌所致AYLL存在下降趋势，提示厦门市居民肺癌死亡存在老龄化趋势，而PYLLR存在上升趋势，主要是人群肺癌死亡率不断上升引起。本次调查还发现，女性居民AYLL高于男性，提示女性因肺癌导致的早死现象比男性更严重；而PYLL、PYLLR男性高于女性，说明肺癌对男性全人群寿命损失的影响大于女性，降低人群死亡率对于减少男性居民PYLLR意义更大，而早期发现肺癌并积极防治对减少肺癌对女性居民寿命损失的影响作用更明显。

GM（1, 1）模型已被广泛应用于慢性病死亡率的预测^[8]^。本研究用GM（1, 1）模型对厦门市居民肺癌死亡率进行预测，预测值与实际值的平均相对误差仅3.45%，说明拟合效果较好、精度较高，可用此模型对厦门市居民肺癌死亡率进行短期预测。根据预测结果，未来5年厦门市男女肺癌死亡率都将继续升高，且女性升高幅度高于男性，提示肺癌防控形势将越来越严峻，采取有效防治措施以遏制厦门市肺癌死亡率不断上升的态势乃当务之急。本文尝试用GM（1, 1）模型对厦门市居民因肺癌所致AYLL进行预测，模型均合格，MAPE也在可接受范围，提示可用此模型对厦门市居民肺癌死亡所致AYLL进行预测。根据预测结果，未来5年厦门市肺癌所致AYLL将由下降转为上升趋势，2019年将比2014年增加0.85年，提示肺癌造成的早死现象将有所加剧。随着观察时间的延长，模型需要不断加进最新的数据重新拟合，以提高预测精度^[8]^。

总之，近十年来厦门市肺癌死亡率逐年升高，未来肺癌死亡率和人均减寿数都有上升趋势，控制肺癌死亡率迫在眉睫。我们应积极开展控烟宣传，以城市、男性、老年人群作为重点宣教对象，改变其不良生活习惯，积极治理环境污染，并加强肺癌的发病和死亡监测。
